# Relationship between developmental canal stenosis and surgical results of anterior decompression and fusion in patients with cervical spondylotic myelopathy

**DOI:** 10.1186/s12891-015-0728-6

**Published:** 2015-09-28

**Authors:** Jing Tao Zhang, Lin Feng Wang, Yue Ju Liu, Jun Ming Cao, Jie Li, Shuai Wang, Yong Shen

**Affiliations:** Department of Spinal Surgery, The Third Hospital of Hebei Medical University, No. 139 Ziqiang Road, Shijiazhuang, Hebei 050051 People’s Republic of China; Orthopedic Trauma Center, The Third Hospital of Hebei Medical University, Shijiazhuang, Hebei People’s Republic of China

**Keywords:** Anterior decompression and fusion, Cervical spine, Developmental stenosis, Myelopathy

## Abstract

**Background:**

Anterior cervical decompression and fusion (ACDF) has long been the preferred treatment for cervical spondylotic myelopathy (CSM). However, few studies have focused on surgical results of CSM in patients with developmental canal stenosis (DCS). The purpose of this study was to investigate DCS as a comorbidity in patients with CSM and the correlation between surgical results and DCS.

**Methods:**

From January 1995 to December 2005, 122 patients treated with ACDF for CSM were enrolled in this retrospective study. Pavlov’s ratio was used to evaluate cervical spinal canal size, with a value of < 0.82 at least one level indicating DCS. Patients were divided into two groups: those with DCS preoperatively (DCS group, *n* = 50 [41.0 %]) and those without DCS (non-DCS group, *n* = 72). Clinical data and radiological parameters were compared between groups.

**Results:**

There were no significant differences in preoperative and 2-year follow-up Japanese Orthopedic Association scores between groups. Both groups achieved satisfactory fusion rates (DCS, 92.0 %; non-DCS, 93.0 %). Adjacent-segment degeneration (ASD) was detected in 66.0 % of patients in the DCS group and in 43.0 % of patients in the non-DCS group (*p* = 0.01). However, there was no significant difference in the incidence of ASD requiring surgery between groups (*p* = 0.20).

**Discussion:**

DCS is a common comorbidity in patients with CSM. The findings of this study have added knowledge on the correlation between DCS and ASD after anterior fusion surgery.

**Conclusions:**

DCS did not affect neurologic improvement postoperatively at short-term follow-up. Although DCS increased the incidence of ASD after anterior fusion, it did not predict ASD requiring surgery. Therefore, patients with DCS must receive close follow-up.

## Background

Cervical spondylotic myelopathy (CSM) is a common disease in older people. Its etiology is multifactorial and includes degenerative changes and/or instability of cervical spine. CSM patients have a high incidence of developmental canal stenosis (DCS), and DCS alone may predispose CSM patients to myelopathy. Anterior cervical decompression and fusion (ACDF) can decompress the spinal cord anteriorly and preserve the stability of the spinal column. However, few studies have investigated the incidence of DCS and surgical outcome of CSM in patients with DCS [1–3]. The purpose of the present study was to investigate morbidity from DCS among CSM patients who underwent ACDF in our hospital. Furthermore, we compared the surgical outcomes of ACDF between CSM patients with and those without DCS to determine whether cervical spine DCS, as a static factor of the cervical spine, influenced surgical outcomes.

## Methods

### Patients

From January 1995 to December 2005, 186 patients with CSM (excluding myelopathy caused by cervical ossification of the posterior longitudinal ligament, trauma, tumor, or infection) underwent ACDF at our hospital. Operative indications for CSM patients were as follows: (1) Up to three levels of anterior cord compression: ACDF; (2) more than three levels of anterior cord compression: laminoplasty; and (3) anterior and posterior cord compression: laminoplasty with or without ACDF. If cord compression was not limited to the level of the disc space but extended over the entire vertebra, cervical corpectomy was indicated for decompression. Based on these indications, we performed ACDF even in case of preoperative DCS. Furthermore, we excluded patients with a history of posterior decompression or whose follow-up periods were less than 5 years. Thus, 122 patients (76 males, 46 females; mean age 61.1 years [range, 46–77 years]) were retrospectively enrolled in this study. Mean duration of myelopathic symptoms prior to operation was 35.9 months, and mean follow-up period was 9.5 years (range, 5–16 years).

The study was approved by institutional review board of the Third Hospital of Hebei Medical University in China and patient consent was waived due to the retrospective nature of this study. Patient information was anonymized and re-identified prior to analysis.

### Surgical technique

After the patient was placed under general anesthesia and positioned supine with the neck slightly extended, the cervical spine was exposed through a standard right-side anterior approach. After complete discectomy and osteophytectomy were carried out, the endplate cartilage was symmetrically removed with a high-speed drill and curette until bleeding occurred. If cord compression was not limited to the level of disc space, but extended over the entire vertebra, subtotal spondylectomy was performed. In all cases, adequate decompression of the cervical spinal cord and the nerve root origin was obtained. After confirming good pulsation of the thecal sac, an appropriate tricortical autograft or corticocancellous allograft was shaped into lordosis and inserted into the intervertebral space. We have previously performed anterior fusion without plating. In addition to this procedure, we subsequently used plating after insertion of the bone graft. In recent years, a titanium mesh cage or polyetheretherketone (PEEK) cage filled with cancellous bone graft has been inserted into the intervertebral space, after which anterior plating is performed. After surgery, a Philadelphia cervical orthosis was applied for 4 weeks and the patient was instructed to wear a soft cervical collar for an additional 2 weeks.

### Radiologic evaluation

**Fig. 1:**
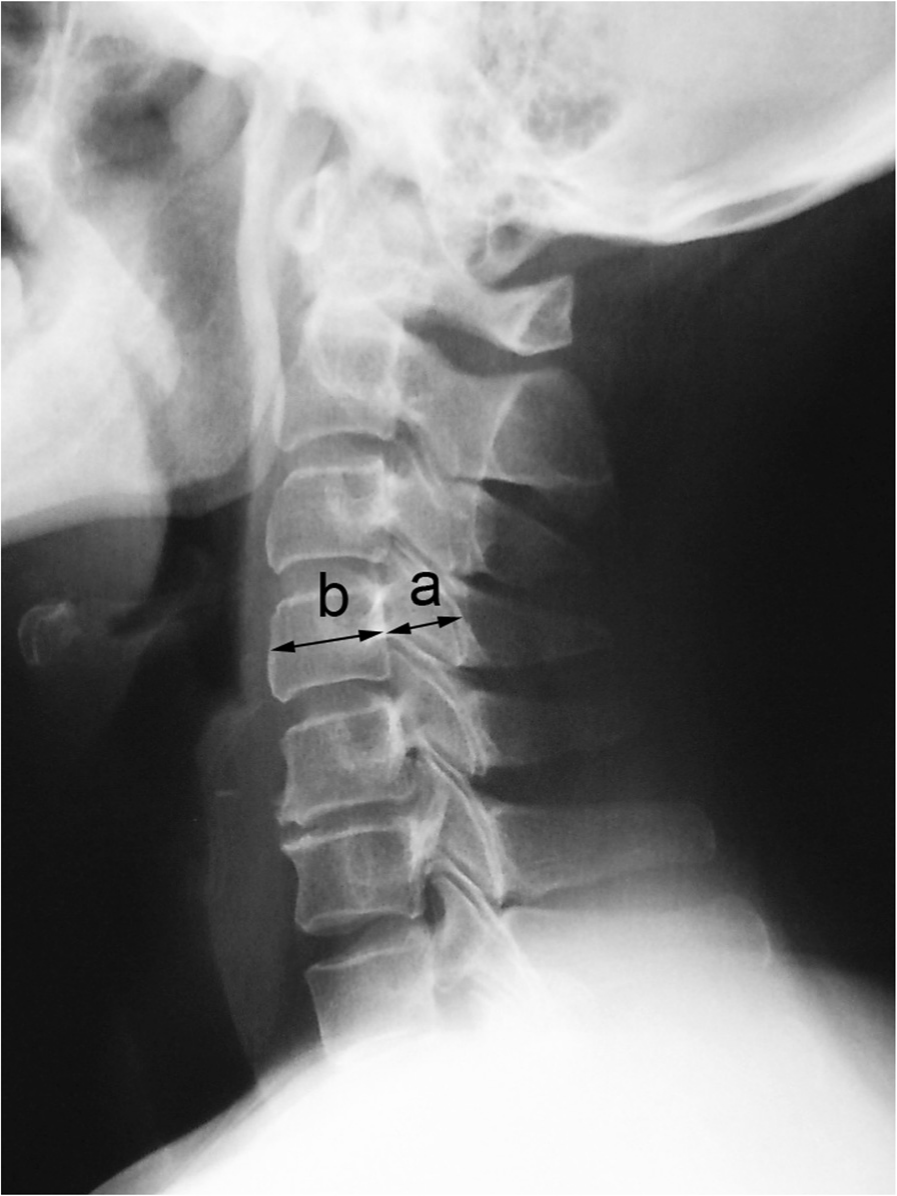
Determination of Pavlov’s ratio. The anteroposterior diameter of the cervical canal **a** is measured from the middle portion of the posterior cortical surface of the vertebral body to the innermost cortical surface of the respective lamina. The anteroposterior diameter of the vertebral body **b** is measured at the midpoint between the anterior surface and the posterior surface. Pavlov’s ratio is determined with the formula a/b. The normal ratio is approximately 1.00

The X-ray beam was focused at C5, and radiographs were all taken at a film-focus distance of 1.5 m. The anteroposterior (AP) diameters of the cervical canal were measured on lateral radiographs between the middle portion of the posterior cortical surface of the vertebral body and the innermost cortical surface of the respective lamina at each vertebral level in neutral position. The sagittal diameter of the vertebral body was measured at the midpoint between the anterior and posterior surfaces. Pavlov’s ratio, which is unaffected by magnification error, was calculated as the sagittal diameter of the cervical canal divided by the sagittal diameter of the vertebral body (Fig. 1). A value of < 0.82 at one level at least indicated DCS [4], and on this basis the 122 patients in this study were classified into two groups: a DCS group and a non-DCS group. The Cobb angle, which was measured between intersecting lines drawn perpendicular to the bottom of the C2 vertebral body and that of the C7 or C6 body on lateral radiographs, was used to evaluate cervical lordosis. Cobb angles of > 10°, 0–10°, and < 0° were judged to be lordotic, straight, and kyphotic, respectively. Osseous fusion was determined 6 months after surgery and was defined on plain radiographs as the absence of lucency around the graft and the presence of bridging bone incorporating the bone graft. A magnetic resonance imaging (MRI) scan of the cervical spine was obtained for each patient. Midsagittal T2-weighted images were assessed by an experienced orthopedic surgeon (author J.L.), who was blinded to patients’ clinical status. Adjacent-segment degeneration (ASD) was determined using modified Hilibrand criteria [5], which were divided into four stages according to plain radiography and MRI. Adjacent segments were rated as having no disease (grade I), mild disease (grade II), moderate disease (grade III), or severe disease (grade IV). Stages II, III and IV were considered indicative of ASD.

### Clinical assessment

Clinical outcomes were evaluated using the Japanese Orthopaedic Association (JOA) scoring system preoperatively and 2 years postoperatively. Using the JOA score, a recovery rate was calculated according to the follow formula: (postoperative JOA score − preoperative JOA score)/(17 − preoperative JOA score) × 100 % [6].

### Statistical analysis

Differences between the DCS and non-DCS groups were evaluated using chi-square analysis and Student’s *t* test. A *p* value < 0.05 was considered statistically significant.

## Results

### Clinical outcomes

According to our classification, the DCS group and the non-DCS group comprised 50 and 72 patients, respectively. There were no significant between-group differences in patient age, sex, and duration of symptoms (Table 1). Ninety-eight patients underwent surgery with the Smith-Robinson procedure [7], and 24 underwent subtotal spondylectomy. In total, 226 disc lesions or spondylotic osteophytes were excised and fused. Forty-five patients underwent single-level fusion, 50 had two-level fusion, and 27 had fusion of three levels. There were no significant differences observed between DCS group and non-DCS group in fused levels (*p* = 0.40).

**Table 1:** Demographics and preoperative data in patients with CSM

	DCS group	Non-DCS group	*p* value
Age (yrs)	61.3 ± 5.6	61.0 ± 7.1	0.74
Sex (Male: Female)	35: 15	41: 31	0.14
Duration of symptoms (mos)	38.4 ± 20.7	34.2 ± 23.6	0.31
FU (yrs)	9.8 ± 2.8	9.1 ± 2.6	0.23
Lordotic angle (°)	8.0 ± 9.9	9.5 ± 15.0	0.54
Minimum AP diameter of cervical canal (mm)	12.0 ± 0.7	14.8 ± 1.1	<0.01
Disc spaces operated (No. of patients)			
1 level	15	30	0.40
2 levels	22	28	
3 levels	13	14	

FU indicates follow-up

AP indicates anteroposterior

Table 2 compares preoperative JOA scores for the DCS and non-DCS groups. Table 3 shows JOA scores and recovery rate at 2-year follow-up for both groups. There were no significant differences in preoperative and 2-year follow-up JOA scores between groups (*p* = 0.11 and *p* = 0.13).

**Table 2:** Preoperative JOA scores in patients with CSM

	DCS group	Non-DCS group	*p* value
Motor function			
UEs	2.0 ± 0.7	2.1 ± 0.8	0.28
LEs	1.3 ± 0.4	1.4 ± 0.6	0.14
Sensory function			
UEs	0.6 ± 0.2	0.6 ± 0.1	0.51
LEs	1.0 ± 0.5	1.1 ± 0.4	0.10
Trunk	1.2 ± 0.7	1.3 ± 0.6	0.33
Bladder function	1.3 ± 0.3	1.4 ± 0.3	0.11
Total	7.4 ± 1.5	7.9 ± 2.0	0.11

*UE* indicates upper extremity

*LE* indicates lower extremity

**Table 3:** The JOA scores and recovery rate at 2-year follow-up in patients with CSM

	DCS group	Non-DCS group	*p* value
Motor function			
UEs	3.2 ± 0.3	3.3 ± 0.5	0.26
LEs	2.3 ± 1.0	2.5 ± 0.8	0.42
Sensory function			
UEs	0.9 ± 0.5	1.0 ± 0.6	0.25
LEs	1.3 ± 0.6	1.4 ± 0.6	0.27
Trunk	1.8 ± 0.2	1.8 ± 0.7	0.37
Bladder function	2.5 ± 0.6	2.6 ± 0.4	0.71
Total	12.0 ± 1.7	12.6 ± 1.9	0.13
Recovery rate (%)	49.9 ± 10.8	53.0 ± 13.0	0.18

*UE* indicates upper extremity

*LE* indicates lower extremity

### Radiological findings

The minimum cervical canal AP diameter was 12.0 ± 0.7 mm in DCS group and 14.8 ± 1.1 mm in non-DCS group (*p* < 0.01). Lordotic angles did not differ significantly between groups either preoperatively (*p* = 0.54) or at final follow-up (*p* = 0.58) (Tables 1 and 4).

**Table 4:** Postoperative data at final follow-up in patients with CSM

	DCS group	Non-DCS group	*p* value
Lordotic angle (°)	13.6 ± 10.6	15.0 ± 12.8	0.58
Fusion rate ※ (%)	92.0	93.0	0.54
ASD	33/50 (66.0 %)	31/72 (43.0 %)	0.01
ASD-S	8/50 (16.0 %)	6/72 (8.3 %)	0.20

※Fusion rate at 6 months postoperatively

*ASD* indicates adjacent segment degeneration

*ASD-* S indicates adjacent segment degeneration requiring surgery

**Fig. 2:**
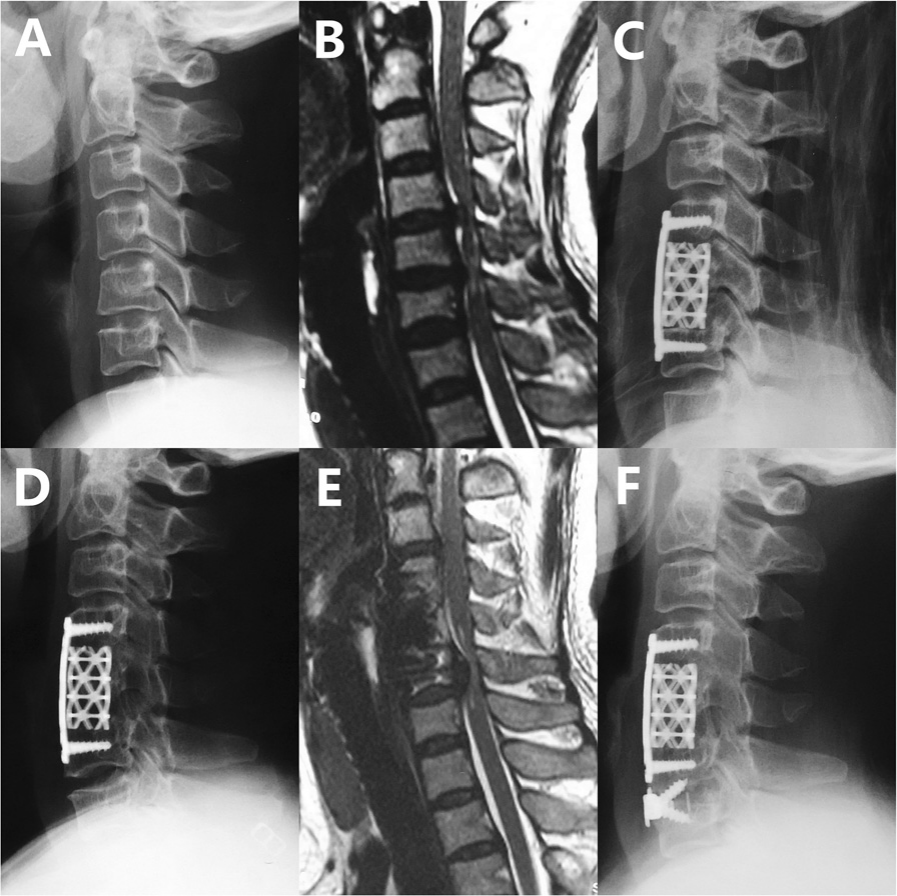
C4 to C7 DCS with recurrent myeloradiculopathy 6 years after C4-C6 anterior cervical decompression and fusion (ACDF). **a** Preoperative radiograph shows DCS at C4-C7 levels. **b** Preoperative MRI of this 49-year-old female shows severe compression of the spinal cord at C4-C6 levels. **c** Radiograph after initial surgery shows ACDF at C4-C6. **d** Radiograph 6 years after initial surgery shows osseous fusion of C4 and C6 and development of adjacent-segment degeneration (ASD) at C6-7. **e** MRI at 6-year follow-up shows development of ASD and spinal cord compression at C6-7. **f** Radiograph after the second surgery shows ACDF at C6-C7. ACDF, anterior cervical decompression and fusion; ASD, adjacent-segment degeneration; DCS, developmental canal stenosis, MRI, magnetic resonance imaging

**Fig. 3:**
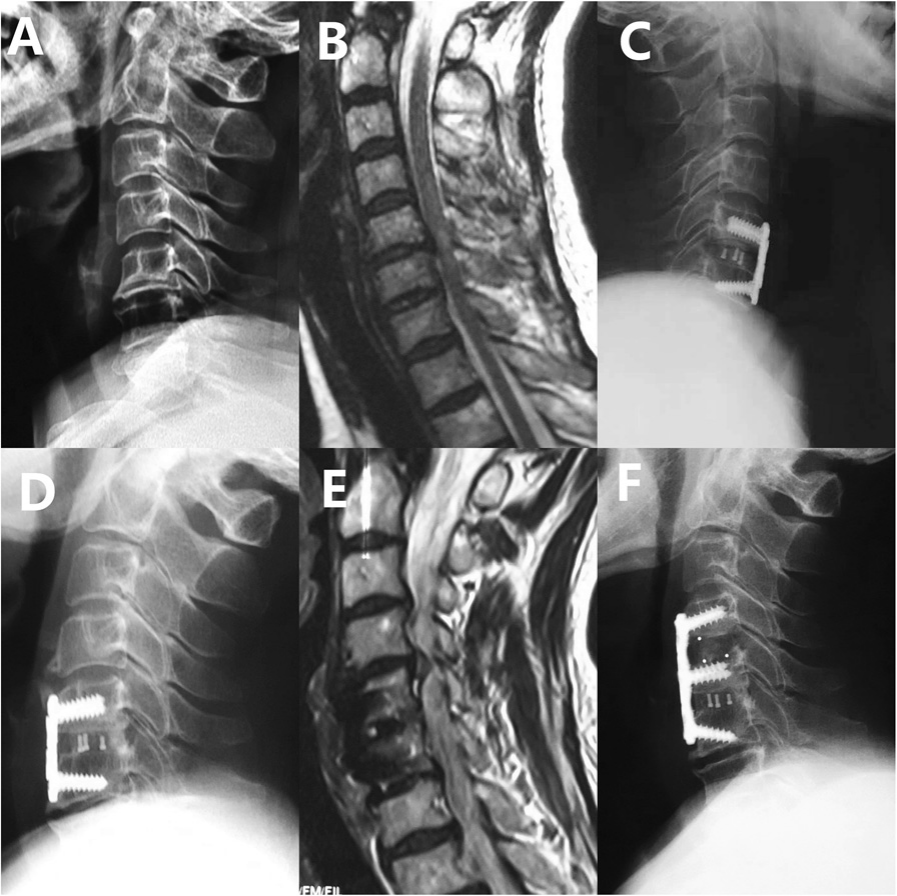
Non-DCS patient with recurrent myelopathic symptoms 8 years after anterior fusion of C5 to C6. **a** Preoperative radiograph shows non-DCS at any level. **b** Preoperative MRI of this 55-year-old female shows severe compressions of the spinal cord at C5-C6 level as well as high signal intensity in the spinal cord. **c** Radiograph after initial surgery shows ACDF at C5-C6. **d** Radiograph 8 years after initial surgery shows osseous fusion of C5 to C6 and development of ASD at C4-C5. **e** MRI at 8-year follow-up shows development of ASD and spinal cord compression at C4-C5. **f** Radiograph after the second surgery shows ACDF at C4-5. ACDF, anterior cervical decompression and fusion; ASD, adjacent-segment degeneration; DCS, developmental canal stenosis, MRI, magnetic resonance imaging

ASD was detected in a significantly greater proportion of patients in the DCS group than in the non-DCS group (66.0 % vs. 43.0 %, *p* = 0.01; Figs. 2 and 3). Fourteen patients required additional surgery after initial ACDF because of symptoms consistent with ASD. All 14 patients had a period of neurologic improvement postoperatively but presented with recurrent myelopathy and/or radiculopathy caused by adjacent segment pathology at follow-up. However, ASD requiring surgery (ASD-S) developed in 16.0 % of patients (8/50) in the DCS group and in 8.3 % of patients (6/72) in the non-DCS group, with no significant difference between groups (*p* = 0.20). At the second surgery, anterior fusion was performed in 4 patients, and 8 underwent posterior surgery. Two patients declined a second surgery because of old age. The average period from the initial ACDF to the occurrence of ASD-S was 8.9 years (range, 3–15 years) (Table 4).

## Discussion

The aging process causes degenerative changes in the cervical spine. Osteophytes and narrowed intervertebral discs are common degenerative changes in the cervical spines of elderly people. Cervical myelopathy is a serious form of cervical spondylosis among those aged over 55 years [8]. Although the exact pathophysiology of cervical myelopathy remains unclear, it is widely accepted that this disorder involves compressive forces on the cervical spine, likely caused by multiple factors. As a static mechanical factor, developmental spinal canal stenosis places a patient at increased risk for developing cervical cord compression and myelopathy. Numerous authors have identified the normal AP diameter of the cervical spinal canal to be approximately 17–18 mm from C3 to C7 [9–11]. However, patients with a sagittal diameter <13 mm are at increased risk for developing signs and symptoms of cervical myelopathy [12]. To avoid magnification variables, Pavlov described the canal-to-body ratio as a reliable determinant of cervical spinal stenosis. A value > 1 is regarded as normal; a value < 0.82 indicates DCS [4].

In this study, the diameters of the spinal canal and vertebral body were measured using plain radiographs because the posterior longitudinal ligament could not be distinguished from the vertebral body on MRI. Although spinal canal size is an important predisposing factor for myelopathy, DCS is not always correlated with neurologic deficit [13]. Hayashi et al. [13] reported that 10 % of subjects over 60 years of age with critical static canal stenosis measuring < 13 mm displayed no neurological deficits. These results suggest that the development of cervical myelopathy may be dependent on other factors in addition to the narrowed spinal canal.

Mechanically, myelopathy has been directly attributed to morbid processes such as spondylotic encroachment on the cervical spinal canal. Nevertheless, a narrowed spinal canal, as a second etiological factor, is thought to allow insufficient space to accommodate an average amount of spondylotic encroachment. In our study, DCS was recognized in 41.0 % of all the enrolled patients. Although DCS is a very important factor influencing the development of cervical myelopathy, it did not affect the clinical results in the current study, and there was no difference in the pre- and postoperative JOA scores or in the recovery rate between DCS and non-DCS groups.

Cervical myelopathy patients with a developmentally narrow canal may have either DCS with no significant evidence of spondylosis or DCS with clear evidence of spondylosis causing pressure on the cervical spinal cord. Epstein et al. [14] felt that in the former situation, decompressive laminectomy should be performed. However, according to Kadoya et al. [15], few cases of myelopathy are caused by the narrowed spinal canal itself; instead, they are attributed primarily to spondylotic changes with advancing age. Therefore, Kadoya et al. [15] emphasized that in the latter case, ACDF should be performed and laminectomy will consequently not be required. Several authors argue that the enlargement of the spinal canal by anterior decompression is limited to surgically repaired segments [16–18], and postoperative deterioration of myelopathy due to the development of instability and/or bone spurs at adjacent levels may be more likely to occur in a narrowed spinal canal [16, 18]. Baba et al. [19] observed that 25 % of patients undergoing anterior cervical fusion subsequently developed new spinal canal stenosis above the fused segments over an average of 8.5 years of follow-up. Bohlman et al. [20] reviewed 122 patients after ACDF with an average of 6 years of follow-up and observed that 9 % of all patients developed ASD-S. Morishita et al. [21] found that a congenitally narrow canal had different effects on cervical kinematics. In other words, subjects with a congenitally narrow cervical canal may be exposed to large mechanical loading at the cervical spine. Their results suggested that a cervical spinal canal diameter of <13 mm may be a risk factor for degenerative disc disease as measured on MRI studies. Eubanks et al. [22] found that although congenital stenosis increases the incidence of radiographic ASD, it does not appear to predict symptomatic ASD. In the present study, the incidence of ASD after ACDF was significantly higher in the DCS group, but the incidence of ASD-S did not differ between groups.

The achievement of optimal alignment is a major goal of cervical spine surgery. Generally, kyphosis might be a contraindication for laminoplasty decompression in CSM patients because kyphosis cases might be insufficient for the posterior shift of the spinal cord. However, ACDF can achieve good release and distraction, so an improvement of cervical alignment can be achieved and maintained. Our radiological outcomes support previous results [23, 24], which show a significant improvement of cervical lordosis.

It is well known that failure of fusion remains a major limitation of multilevel ACDF. In our study, 4 patients in the DCS group and 5 in the non-DCS group exhibited non-fusion 6 months after operation. This finding is consistent with prior reports. In 2002, Barnes et al. [25] reported satisfactory outcomes in only 65 % of patients who had undergone multilevel ACDF, with an overall fusion rate of only 90 %.

## Conclusions

In summary, we evaluated the clinical significance of DCS in CSM patients treated with ACDF. DCS was observed in 41.0 % of our CSM patients. DCS can greatly influence CSM, but it did not affect neurologic improvement postoperatively at the short-term follow-up. Although DCS increased the incidence of ASD after anterior fusion, it did not predict ASD-S. Therefore, patients with DCS must receive close follow-up.

## Abbreviations

ACDF: Anterior cervical decompression and fusion
CSM: Cervical spondylotic myelopathy
DCS: Developmental canal stenosis
ASD: Adjacent segment degeneration
ASD-S: Adjacent segment degeneration requiring surgery
AP: Anteroposterior
MRI: Magnetic resonance imaging
JOA: Japanese Orthopaedic Association
FU: Follow-up
UE: Upper extremity
LE: Lower extremity
